# The Treatment of Organic Stricture of the Uerthra by Combined Internal and External Urethrotomy with Perineal Drainage

**Published:** 1891-08

**Authors:** F. W. McRae

**Affiliations:** Demonstrator of Anatomy and Lecturer on Genito-Urinary and Clinical Surgery, Atlanta Medical College, Atlanta, Ga.


VOL. VIII. AUGUST, 1891. NO. 6.

### THE TREATMENT OF ORGANIC STRICTURE OF THE URETHRA, BY COMBINED INTERNAL AND EXTERNAL URETHROTOMY WITH PERINEAL DRAINAGE.*

BY F. W. MCRAE, M. D.,
Demonstrator of Anatomy and Lecturer on Genito-Urinary and Clinical Surgery, Atlanta Medical College, Atlanta, Ga.

### REPORT OF CASES.

In the discussion of this subject it is my purpose to confine myself to the consideration of organic strictures of the male urethra not safely curable by simpler methods of treatment. I shall make no allusion to uncomplicated strictures of the penile urethra, nor to those strictures of the deep urethra which may be readily relieved by gradual dilatation.

*Read by caption before Surgical Section of American Medical Association, at Washington (May 1891).

That under many conditions external urethrotomy alone, or combined internal and external urethrotomy, are much safer operations than simple dilatation, divulsion or internal urethrotomy, most genito-urinary surgeons are agreed. The conscientious surgeon must, however, meet with cases of stricture which will puzzle him to decide what method of treatment is safest and is most apt to cure the stricture or give permanent relief to the distressing symptoms which it produces.

The literature on the treatment of organic stricture of the deep urethra is so confusing, and the opinions of equally eminent surgeons are so divergent, that the young surgeon is often at a loss to know just how to proceed or who is the safest guide to follow.

There are, for instance, a few eminent surgeons who practice divulsion or internal urethrotomy for the relief of deep strictures, while many equally eminent surgeons condemn these procedures as unscientific, unsurgical and dangerous. And it is with the latter that my observation and experience have led me to agree. About two per cent. of all internal urethrotomies prove fatal, and the surgeon who assures his patient that the operation is devoid of danger must be either ignorant of the facts or willing to deceive.

That these operations are much safer when done according to the rules of strict antisepsis, goes without saying, but that antisepsis makes them safe procedures under all circumstances I think no one will assert.

The dangers to which divulsion and internal urethrotomy especially subject the patient are urethral fever, extravasation of urine and hæmorrhage. Though the danger of urethral fever decreases in proportion to the more nearly the urine maintains a normal standard, other things being equal, we cannot positively prevent its occurence after these operations, by any known prophylactic measures.

Where, however, the stricture is of long standing, small calibre, irritable, risilient and complicated by cystitis, with ammoniacal urine, the dangers are very greatly enhanced. Given such a case in an old or enfeebled individual and the result of either divulsion or internal urethrotomy would be, in a large proportion of cases, death, though the operation be done according to the most approved technique.

I do not wish to be understood as arguing against internal urethrotomy in properly selected cases. Nor do I believe divulsion should be universally condemned.

Were it not for the fact that we have better methods of treatment most strictures might be relieved by one of these operations. The question is how are we to relieve those tight, irritable, complicated strictures with greatest safety to the patient and secure the greatest immunity from return ? It seems to me the proper answer is, "by combined internal and external urethrotomy with perineal drainage."

To Sir Reginald Harrison belongs the credit of clearly demonstrating the great superiority of this method of treatment over all others in the class of cases to which we have alluded, even where the strictures are readily passable. He has done the combined operations in considerably over an hundred cases without a fatal result or serious complication.

The subsequent histories of these cases were much freer from complications than were those of patients operated upon by either divulsion or internal urethrotomy, and the results more lasting. Mr. Harrison has not met with a single case of return of stricture after this operation. My own experience, though comparatively limited, coincides with his in so far as my observation has extended. As illustrative, I append brief reports of three cases I have recently operated upon according to his method.

CASE I.—Mr. K., *aet* 48, married, engineer. History of repeated attacks of gonorrhœa, cured with greatest difficulty. Since marriage he has had discharge from urethra, often lasting for months, and which for the last year or two has been almost continuous. Was treated for stricture about five years ago by gradual dilatation. Left off using bougies as directed. Several recent attempts—twice under anæsthesia—to pass instrument into bladder had failed. Frequent urination day and night. Urine S. G. 1012 alkaline, muddy, trace of albumen. Deposit contained abundance of pus, bladder epithelium and phosphates. No casts or kidney epithelium.

Operation St. Joseph's Infirmary (January 11, 1891), ether. After repeated attempts, extending over an hour and a half, succeeded in passing filliform through stricture. Impossible to pass guide over filliform. Operation completed without very great difficulty. Introduced No. 30 soft rubber catheter through perineal wound, to which was attached rubber tube of sufficient length to conduct urine into a receptacle on floor beside the bed. Antiseptic dressing held in place by T bandage. Strict antisepsis throughout. Patient bore operation exceedingly well. Subsequent history, afebrile and without complication. Catheter removed eighth day. Sounds introduced every four to seven days. Patient left the city three weeks after operation, perineal wound almost closed. Passes 32 steel sound without difficulty. When last heard from there was still little escape of urine through perineal opening, condition otherwise excellent. [Later. Perineal opening closed.]

CASE II.—G. K., colored, *aet* 52, married, had gonorrhœa when about twenty, not since. History of difficult urination, with occasional fits of retention, extending over a period of several years. On examination, found one old perineal fistula and scars where others had closed. Tortuous stricture in penile urethra, tough, fibrous in character ; dense stricture of small calibre just posterior to bulb, numerous false passages.

Operation before class Atlanta Medical College, January 28th. Ether. Impossible, after repeated efforts, to introduce staff or filliform; always caught in false passages. Finally succeeded in passing No. 10 gum elastic catheter. Operation very tedious. Hæmorrhage excessive; controlled by introducing catheter and packing around with iodoform gauze. Dressed as case I. Catheter removed third day. No subsequent hæmorrhage. Perineal wound completely healed in four weeks.

CASE III.—Mr. D., fireman, *aet* 38, single. Fell astride buggy wheel when fourteen years old, bruising perineum. Had gonorrhœa in 1868, 1875 and 1890. Urinates with greatest difficulty and at frequent intervals. Occasional chills. On examination, found two strictures of large calibre in penile urethra and one dense stricture of small calibre, only admitting filliform, in membranous urethra. Examination followed by chills and increased difficulty in urinating. Subsequent introduction of filliform one week later followed by repeated violent chills.

Operation St. Joseph's Infirmary, February 10th. Ether. Filliform introduced without difficulty, over which succeeded in passing small tunnelled catheter. Operation completed without difficulty. Dressed as in cases I and II. Catheter removed eighth day. Few slight chills after removal of catheter. Recovery excellent. Now passes 34 sound without difficulty, and perineal wound is about closed, rarely allowing escape of a few drops of urine.

While all of the cases reported might possibly have been relieved by gradual dilatation or divulsion, I am sure they could not have been relieved so quickly, safely or pleasantly by either of those operations.

There is, of course, the remote possibility in every case of producing a perineal fistula, but the probability is so slight as to weigh very little with either patient or physician. Where the strictures have been thoroughly cut the danger of not closing is almost *nil.* I am sure that perineal drainage was of the greatest value in these cases, and that it possesses all the advantages Mr. Harrison claims for it. Patients are rendered much more comfortable by the tube. There is very little danger of its causing cystitis unless it is introduced too far. The bladder should be washed out at least once daily with some mild antiseptic solution. The urine being prevented from coming in contact with the cut and abraded tissues, the danger of septicemia or of urethral fever is reduced to a minimum. Where there is a chronic discharge, the urethral mucous membrane is given complete rest and it is allowed to get well. My results with this operation have been so much more favorable than I have been able to obtain by other methods of treatment that I feel like it should be adopted in the treatment of all organic strictures of the deep urethra not readily amenable to dilatation.

